# Toxicity Effect of Silver Nanoparticles on Photosynthetic Pigment Content, Growth, ROS Production and Ultrastructural Changes of Microalgae *Chlorella vulgaris*

**DOI:** 10.3390/nano9070914

**Published:** 2019-06-26

**Authors:** Layla J. Hazeem, Gamze Kuku, Etienne Dewailly, Christian Slomianny, Alexandre Barras, Abderrahmane Hamdi, Rabah Boukherroub, Mustafa Culha, Mohamed Bououdina

**Affiliations:** 1 Department of Biology, College of Science, University of Bahrain, P.O. Box 32038, Zallaq 1054, Bahrain; 2 Department of Genetics and Bioengineering, Yeditepe University, Atasehir 34755, Istanbul, Turkey; 3 Laboratoire de Physiologie Cellulaire, INSERM U.1003, Université de Lille, Rue Paul Langevin, 59655 Villeneuve d’Ascq, France; 4 Univ. Lille, CNRS, Centrale Lille, ISEN, Univ. Valenciennes, UMR 8520-IEMN, F-59000 Lille, France; 5 Department of Physics, College of Science, University of Bahrain, P.O. Box 32038, Zallaq 1054, Bahrain

**Keywords:** silver nanoparticles, Ag ions, *Chlorella vulgaris*, viable cells, chlorophyll *a*, reactive oxygen species

## Abstract

Silver nanoparticles (Ag NPs) exhibit antibacterial activity and are extensively used in numerous applications. The aim of this study was to examine the toxic effect of Ag NPs on the marine microalga, *Chlorella vulgaris.* The microalgae, at the exponential growth phase, were treated with different concentrations of Ag NPs (50 and 100 nm) for 96 h. X-Ray diffraction (XRD) results indicated that the used NPs are single and pure Ag phase with a mean crystallite size of 21 and 32 nm. Ag NPs were found to have a negative effect on viable cell concentration, a variable effect on chlorophyll *a* concentration, and increased ROS formation. Transmission electron microscopy (TEM) analysis revealed that Ag NPs were present inside the microalgae cells and formed large aggregates in the culture medium. Ag^+^ ions, in the form of AgNO_3_, were also assessed at higher concentrations and found to cause inhibitory effects.

## 1. Introduction

Silver nanoparticles (Ag NPs) are extensively used in numerous applications [1]; they are employed in more than two hundred customer manufactured goods, including deodorants and socks [2]. Due to their antibacterial and antiviral properties [3,4], they are utilized for medical purposes, employed in many engineered products, and were added to detergents as dynamic compounds [5,6,7,8,9]. They already have applications in personal care products, food storage boxes, laundry, domestic devices, paints, as well as cooking additives [10]. They can be combined with other materials to demolish harmful algal blooms—for instance, by employing TiO_2_ semiconductors functionalized with Ag NPs [11]. They are also used to control the growth of aquatic plants that behave as lethal aquatic algal weed, often clogging domestic water supply and water ducts [12]. It is significantly important to point out that Ag NPs are produced in different sizes and will therefore have diverse molecular reactivity properties depending on their surface-area-to-mass ratio [13].

Ag NPs, like other nanomaterials, can be easily transferred to water environments [14]. While Ag NPs offer numerous benefits, their overall toxicity is not very well determined. The scarce published studies of the toxic effects of Ag NPs on natural systems state dissimilar and even contradictory outcomes [15]. Jiang et al. [16] showed that Ag NPs and AgNO_3_ could accumulate in aquatic biotic components that might be taken by other higher trophic animals; hence, Ag NPs and AgNO_3_ could be transferred to food webs which infer hazards for human health. According to Kennedy et al. [17], Ag is considered as the second most lethal metal for aquatic creatures, after mercury (Hg). Toxic effects of Ag NPs may possibly be related to damages at cell surfaces, to oxidative stress and formation of reactive oxygen species (ROS), or to contacts of Ag cations with cellular proteins and enzymes [6,18,19]. Moreover, Ag NPs were found to have variations in toxicity effects on different organisms and different experimental conditions, with the lowest recorded No Observed Effect Concentration (NOEC) values of 0.001 µg L^−1^ for *Daphnia sp*. [20]. Though to fully comprehend and assess their impact on aquatic living organisms, more studies and research should be carried out.

In aquatic ecosystems, the microscopic algae, as critical principal producers, are the main target for the majority of contaminants. Due to their role as the first level of the trophic webs, any trouble encountered by them will eventually have a consequence on the remaining of the ecosystem [21].

Algae species, as model organisms to toxicity tests, exhibit diverse responses to various toxic materials [22]. Furthermore, Klaine et al. [13] stated that huge dissimilarities were present in the performance of NPs in seawater when compared to freshwater environments. For instance, it has been reported that salt-based media are capable of dissolving more Ag^+^ from Ag NPs than freshwater media [23,24]. Additionally, the study of the toxic effect of Ag NPs is more complex as both particles and ions can coexist in the media [25]. Likewise, there are conflicting studies emphasizing either silver ions or NPs as the major cause of toxicity [26]. Together, media and the Ag dissolved from Ag NPs cause a vital contribution to the negative effect of metallic NPs in aquatic environments [27]. Thus, the effect on living organisms—including algae—of being exposed to NPs will ultimately vary. For example, Oukarroum et al. [28] demonstrated that Ag NPs have variable effects on the freshwater microalga, *Chlorella vulgaris,* and the marine alga, *Dunaliella tertiolecta*. In another study by Oukarroum et al. [29], it was shown that temperature increases the noxious consequences of Ag NPs on aquatic alga. Total inhibition of algal growth of the freshwater microalga *Pseudokirchneriella subcapitata* was caused by Ag NPs at 5 mg L^−1^ [30]. He et al. [31] found that the toxicity of Ag was mainly due to silver ions. Likewise, Navarro et al. [32] stated that Ag^+^ ions discharged from Ag NPs were toxic to the freshwater *Chlamydomonas reinhardti*.

Several toxicity tests and reviews have revealed that NP size, agglomeration/aggregation, as well as the settlement of these NPs have a significant role in NPs’ toxicity [33,34]. A valuable review article by Moreno-Garrido et al. [35] described a good comparison between the diverse effects induced by Ag NPs (coated and non-coated forms, different sizes) on marine and freshwater microalgae species, though it is apparent that there are fewer studies carried out on marine microalgae.

Marine algae, owing to their great sensitivity to synthetic nanoparticles and high buildup ability, can be used as an indicator of pollution in marine ecosystems [36]. Therefore, more emphasis on the effect of Ag NPs on marine model organisms is required. *Chlorella vulgaris*, the model organism used in the present study, has a cell wall that will enhance the binding between NPs and algal cells. Additionally, the cell wall of microalgae is characterized by pores with thicknesses ranging from 5 to 20 nm [4]. One of the few studies that examined the effect of the Ag NPs size on different organisms was conducted by Angel et al. [37]. They found that the least toxic Ag was the micron-sized, due to the slow rate of dissolution. Burchardt et al. [38] studied the size effect of Ag NPs (20, 40, and 100 nm) on the freshwater cyanophyte, *Synechococcus sp.,* and marine diatom, *Thalassiosira pseudonana,* at different concentrations (0.05–10 µM). The measured EC_50_ values recorded for 72 h incubation were 1.2 µM and 0.9 µM for the diatom and for the cyanophyte, respectively. They demonstrated that both Ag NPs and the free Ag^+^ ions were accountable for the toxicity in the tested organisms. In contrast, Navarro et al. [32] reported that the size of the NPs did not account for variation in toxicity. Other reports showed that smaller Ag NPs were more toxic than larger ones [39,40]. The contradictory results in the literature may indicate that there are several factors influencing the negative effect of Ag NPs, including the test organisms.

The present study aims to examine the effect of Ag NPs of two different sizes (50 and 100 nm) at different concentrations (10, 50, 100 and 200 mg L^−1^) to assess the effect of both size and concentration of Ag NPs. Chlorophyll *a* concentration, viable cell concentration, (ROS) formation, and extra- and intracellular changes in the microalga were examined.

## 2. Materials and Methods

### 2.1. Chlorella vulgaris Culture

The marine microalga *Chlorella vulgaris* (CCAP211/75; Origin: Marine; bottom sample, Loch Linnhe, Argyll, Scotland, UK) was purchased from Culture Collection of Algae and Protozoa, Scottish Marine Institute, UK. It was grown in a sterile f/2 medium. The cells were grown at 18 °C for 12 h:12 h dark:light cycle with a light intensity of 100 µmol m^−2^ s^−1^. Algal samples were used during their exponential growth phase.

### 2.2. Silver Nanoparticles (Ag NPs) Characterization

Ag NPs were purchased from (American Elements, Los Angeles, CA, USA). According to the manufacturer, the diameters of NPs are 50 and 100 nm. A stock suspension of the different concentrations of both Ag NPs was prepared in the culture medium and sonicated before use for 15 min with ultrasonicator (Ultrasonic Cleaner CT ChromTech Model UC-3120B).

X-ray diffraction patterns were recorded using high-resolution Rogaku Ultma VI diffractometer equipped with CuKα radiation source (λ_Cu_ = 1.5418 Å). The measurements were carried using the following conditions: Voltage V = 40 kV, current I = 40 mA, initial angle 2θ = 20°, final angle 2θ = 80°, angle increment 0.04°, and counting time 1 s.

Scanning Electron Microscopy (SEM) images of the films were obtained using Scanning Electron Microscope (ULTRA 55, Zeiss, Oberkochen, Germany) provided with a thermal field emission emitter. In addition, it was equipped with three detectors (EsB Detector with filter grid, high-efficiency In-Lens SE detector, Everhart-Thornley Secondary Electron Detector) and X-ray energy dispersive analysis device (EDX analysis) (Bruket AXS).

Zetasizer^®^ Nano ZSP (Malvern Instrument S.A., Worcestershire, UK) was used to record the average hydrodynamic diameter of NPs. All the batches were diluted to 200 mg L^−1^ in 0.22 µm filtered sea-water (28 PSU (Practical Salinity Unit)), sonicated for 5 min prior to the analysis, and analyzed at 25 °C with the automatic mode every 600 s during the 60 min. The size distribution of the samples (every 10 min) is presented as a plot of relative light intensity scattered by particles (on the Y-axis) against various size classes logarithmically spaced (on the X-axis).

### 2.3. Algal Inhibition Test

A *Chlorella vulgaris* culture was collected during the exponential growth phase (3.3 × 10^5^ cells mL^−1^). The algae were exposed to increasing concentrations (0, 10, 50, 100, 200 mg L^−1^) of 50 and 100 nm Ag NPs and incubated in the laboratory conditions as stated above for 96 h.

### 2.4. Concentration of Photosynthetic Pigment Chlorophyll a

Determination of chlorophyll *a* concentration was carried out following UNESCO protocol [41]. Glass Fiber Filters (GFF) were used to collect 30 mL of each treated algal culture at 24, 48, 72, and 96 h of incubation. 90% acetone was used to extract chlorophyll *a*, followed by quantification by spectrophotometry (Perkin Elmer UV spectrophotometer, Shelton, USA).

### 2.5. Measurement of Viable Cells

Viable algal cell concentration, in the control and exposed samples to Ag NPs, was precisely measured at 24, 48, 72, and 96 h of incubation using a Muse^TM^ cell analyzer (Millipore, Industrial Blvd, Hayward, CA94545, USA).

### 2.6. Determination of Chlorophyll a Concentration and Viable Cells Using Silver Nitrate

Ag NPs of different sizes were found to cause inhibitory effects on the microalga, *Chlorella vulgaris*, at high concentrations (100 and 200 mg L^−1^). Therefore, an experiment was conducted using silver nitrate (AgNO_3_) at 100 and 200 mg L^−1^ to determine if the toxic effect was induced by nanoparticles or released Ag^+^ ions. *Chlorella vulgaris* was incubated with AgNO_3_ in f/2 medium during the exponential growth phase, and both chlorophyll *a* and viable cells were measured at 24, 48, 72, and 96 h. Both chlorophyll *a* concentration and viable cells were measured as mentioned above.

### 2.7. Measurement of Reactive Oxygen Species (ROS) Formation

The level of Reactive Oxygen Species (ROS) formation was determined with DCFDA Cellular ROS Detection Assay Kit (Abcam, catalog no. ab113851), following the manufacturer’s procedure. *Chlorella vulgaris* was aliquoted into vials to contain 1 × 10^5^ cells per mL for each treatment in triplicates. Dispersed 50 and 100 nm Ag NPs were added to each vial at increasing concentrations (10, 50, 100 and 200 mg L^−1^) and incubated for 96 h. Blank samples containing Ag NPs in culture medium were also prepared for each concentration. One hour prior to the completion of the 24, 48, 72, and 96 h time points, 100 µL from each sample was transferred into a 96-well plate in triplicates, and another 100 µL of 2× DCFDA (2′,7′–dichlorofluorescein diacetate) was added to wells. For each specific Ag NP concentration, 2× DCFDA was prepared by using their blank samples. At the end of incubation, the 96-well plate was read on a SpectraMax^®^ Paradigm^®^ Multi-Mode Microplate Reader (Molecular Devices, LLC., CA, USA) with an excitation wavelength of 485 nm and emission wavelength of 535 nm. The ratio of relative fluorescence intensities was calculated between the control and treated wells to blank wells.

### 2.8. Transmission Electron Microscopy (TEM) Analyses

Transmission Electron Microscopy (TEM) was used to observe the ultrastructural changes of *Chlorella vulgaris*. 2.5% glutaraldehyde, prepared in 0.1 M cacodylate buffer and post-fixed in 1% osmium tetroxide in the same buffer, was used to fix cells (control and treated samples). After dehydration with acetonitrile, the pellets were embedded in Epon. Later, a Leica UC7 ultramicrotome was used to cut thin section (90 nm) and collected on 150-mesh copper grids. Sections were observed with a Hitachi H-600 transmission electron microscope (Tokyo, Japan) at 75 kV, after staining with 2% uranyl acetate in 50% ethanol and incubation with a lead citrate solution.

### 2.9. Statistical Analysis

All treatments were carried out in triplicate. Mean and standard deviations were calculated for each treatment. Significant differences among the control samples and algal cells exposed to Ag NPs or AgNO_3_ were determined by analysis of variance (ANOVA) followed by Tukey’s pairwise comparison with Minitab version 17. A significance level of *p* less than 0.05 was used. SigmaPlot 13, SYSTAT, USA, was used to create graphs.

## 3. Results

### 3.1. Characterization of Ag NPs

**Figure 1:**
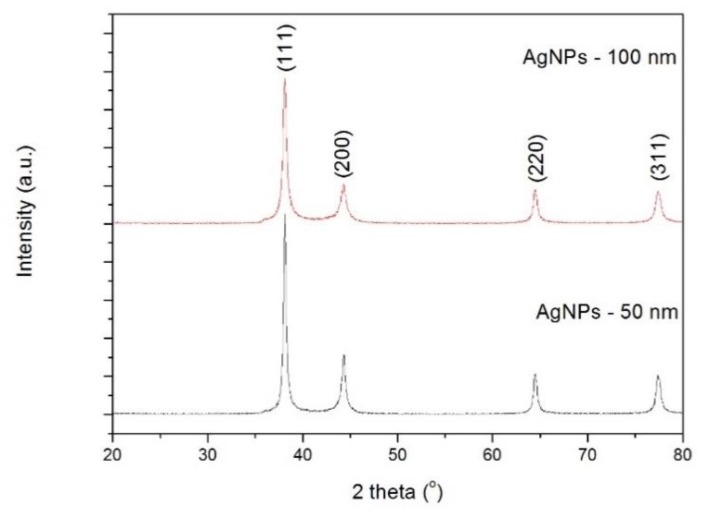
X-ray diffraction patterns of silver nanoparticles (Ag NPs).

Figure 1 displays the X-ray diffraction patterns of 50 and 100 nm Ag NPs. Well defined peaks are observed and indexed within a face centered cubic (FCC) crystal structure, in agreement with JCPDS card No. 04–0783. The crystallite size was estimated using Sherrer’s equation:

(1) $$D=\frac{K\;\lambda }{\beta \cos\theta }$$

where *λ* is the wavelength of the X-ray source, *β* is the full width at half maximum of the (111) diffraction peak and *θ* its corresponding diffraction angle, and *K* a shape factor (for spherical particles *K* ~ 0.9). The calculated crystallite size values are 21 and 32 nm for 50 and 100 nm Ag NPs, respectively. It is known that a particle can be formed by a certain number of small crystals (crystallites). This means that the used Ag NPs are made of a few crystallites.

**Figure 2:**
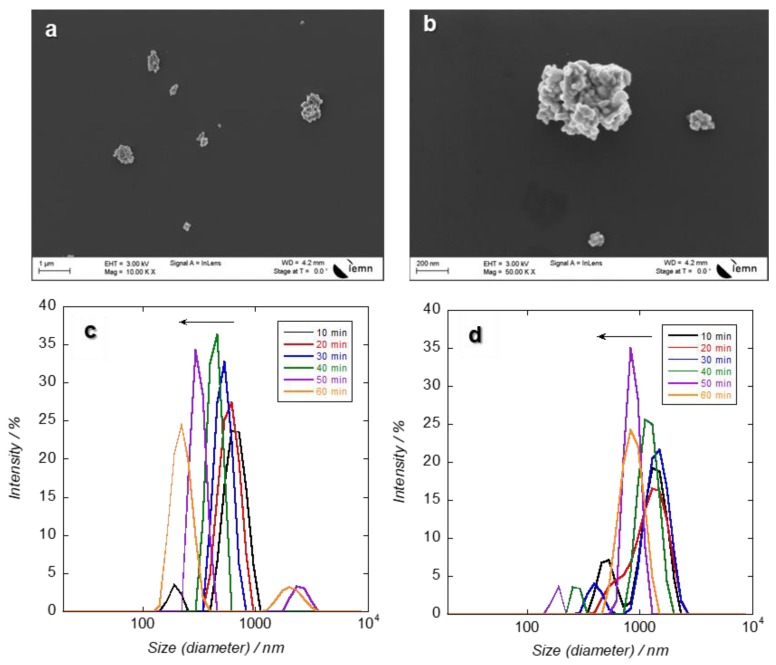
Scanning Electron Microscopy (SEM) images of (**a**) 50 nm and (**b**) 100 nm Ag NPs. Hydrodynamic diameter of (**c**) 50 nm and (**d**) 100 nm Ag NPs in seawater (28 PSU) at 200 mg L^−1^ during 1 h.

The SEM images of the Ag NPs show Ag NPs of irregular shape in agglomerated forms (Figure 2a,b). Ag NPs were found to form large particles and precipitates in the seawater culture medium used in the present study. This is very likely due to the presence of chloride at high concentration in the medium, as f/2 is prepared from seawater and contains a high concentration of chloride ions (salinity recorded was 28 PSU). It is known by now that the high salinity of seawater can cause an increase in dissolved silver with additional aggregation and sedimentation [37]. It was found that agglomerated Ag NPs dispersed well in the medium after sonication but started to precipitate within one hour. Figure 2c,d depicts the evolution of the hydrodynamic diameter of the Ag NPs after dilution in seawater. In seawater, Ag NPs (50 and 100 nm) were found in aggregated forms (549 and 1280 nm, respectively). Just after sonication, the decrease of the derived count rate from successive measurements indicates the particle sedimentation of large agglomerates. The size of aggregates is proportional to the size of the NPs.

Besides that, the pH of the seawater will possibly also affect the aggregation rate influenced by the surface charge of the particles engaged [42]. The initial pH value of the medium was 6.3. After completion of the incubation time (96 h), the pH of the differently treated samples was 6.37 in control and reached maximum values with the 200 mg L^−1^ treated samples using the 50 and 100 nm Ag NPs (6.40 and 6.45, respectively). Since the Ag NPs solutions of different concentrations were prepared in seawater, it is expected that Ag^+^ ions will be discharged by chloride complexing in seawater [35]. It was stated that aggregation of NPs in seawater is further increased when compared to freshwater. Sendra et al. [43] found that dissolution of Ag from Ag NPs was tremendously high in marine water (about 25 times) compared to freshwater.

### 3.2. Concentration of Chlorophyll a

**Figure 3:**
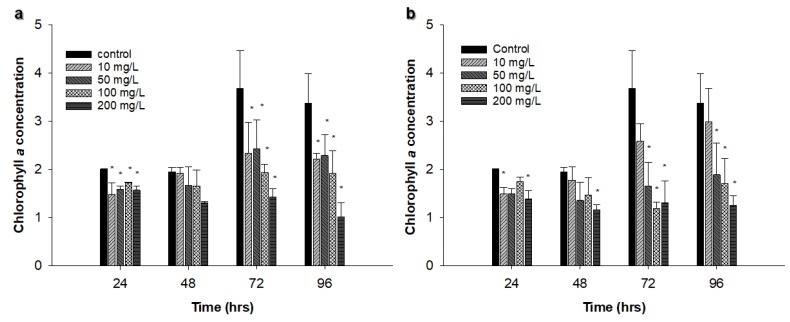
Effect of different concentrations of Ag NPs on *Chlorella vulgaris* chlorophyll *a* concentration in µg L^−1^: (**a**) 50 nm Ag NPs, (**b**) 100 nm Ag NPs. The experiments were performed in triplicate, and results are displayed as the mean with standard deviations (Asterisks specify significant differences from control values (*p* < 0.05); n = 3).

*Chlorella vulgaris*, at the exponential growth phase, was exposed to Ag NPs (50 and 100 nm) at different concentrations (from 10 to 200 mg L^−1^) for 96 h. In the control sample, chlorophyll *a* concentration reached the highest concentration at 72 h (3.68 µg L^−1^). Cells treated with 50 nm Ag NPs showed some fluctuations in chlorophyll *a* concentration during the experiment, and the lowest values were recorded for cells exposed to 200 mg L^−1^ when compared to other treated samples (Figure 3a).

Cells treated with 100 nm Ag NPs exhibited similar trends except cells treated with 10 mg L^−1^ where chlorophyll *a* concentration increased over time. Statistical analysis revealed that there was a significant difference (*p* < 0.05) between control and all treated samples at 96 h using the 50 nm Ag NPs, though there was a significant difference (*p* < 0.05) among control and all treated samples except the 10 mg L^−1^ treated culture at 96 h using the 100 nm Ag NPs (Figure 3b).

### 3.3. Viable Cell Concentration

**Figure 4:**
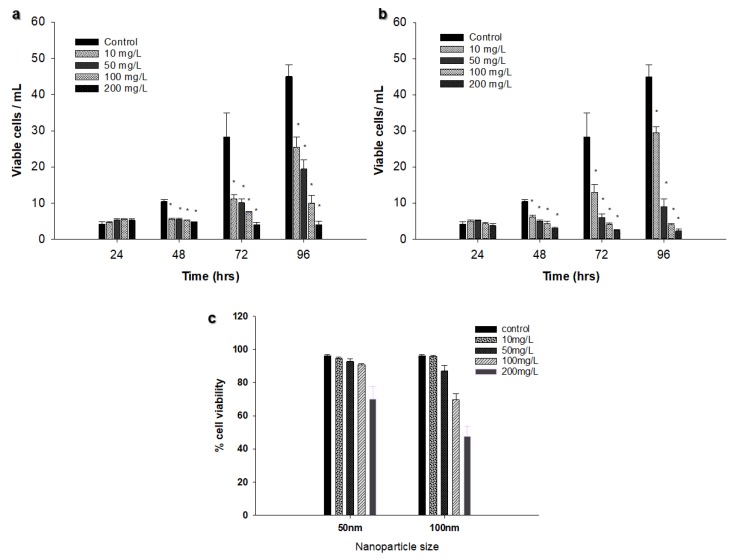
Effect of different concentrations of Ag NPs on viable cell concentration (**a**) 50 nm Ag NPs, (**b**) 100 nm Ag NPs. (**c**) Effect of Ag NPs size and concentration on % of viable *Chlorella vulgaris* cells at 96 h of incubation. The experiments were performed in triplicate, and results are displayed as the mean with standard deviations (Asterisks specify significant differences from control values (*p* < 0.05); n = 3).

There was an increase in viable cell concentration in most samples (control and treated samples) during the 96 h exposure time. Viable cell concentration increased from 4.2 × 10^5^ cells L^−1^ at 24 h to 44.97 × 10^5^ cells L^−1^ at 96 h in the control samples. The only samples that showed a decrease in viable cells were those treated with the 50 nm Ag NPs at 200 mg L^−1^ (from 5.3 × 10^5^ cells mL^−1^ at 24 h to 4.02 × 10^5^ cells mL^−1^ at 96 h) and those treated with 100 nm Ag NPs at 100 mg L^−1^ and 200 mg L^−1^ (3.91 × 10^5^ and 2.29 × 10^5^ cells mL^−1^, respectively at 96 h; Figure 4a,b). A significant difference between control and treatment samples was found at 96 h using both sizes of Ag NPs (*p* < 0.05). However, the only concentration that showed more than 50% reduction in viable cell concentration was 200 mg L^−1^ for the 100 nm Ag NPs (Figure 4c).

**Figure 5:**
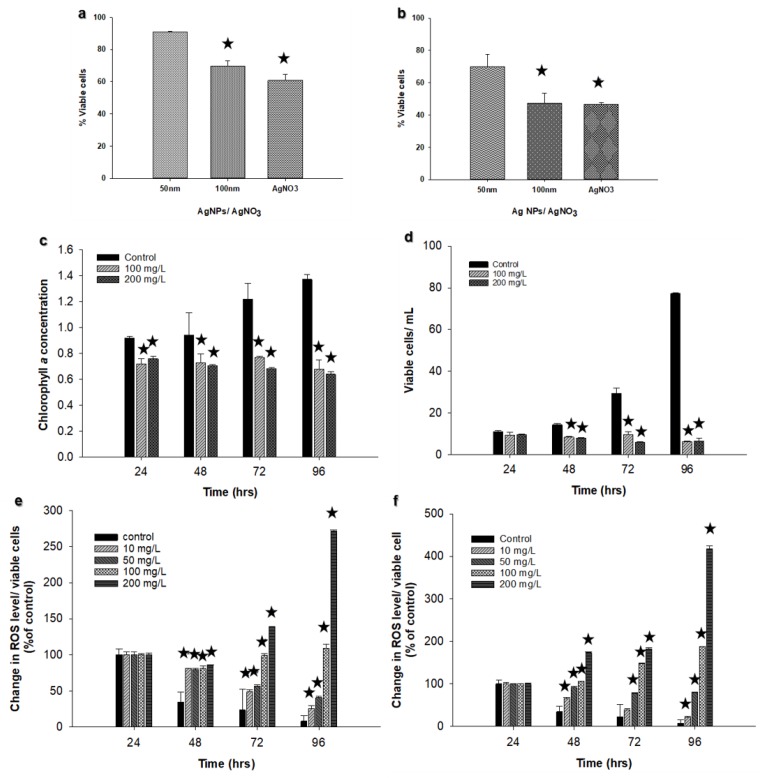
Comparison between Ag NPs and AgNO_3_ at (**a**) 100 mg L^−1^ and (**b**) at 200 mg L^−1^. Effect of AgNO_3_ on chlorophyll *a* concentration in (**c**) µg L^−1^, and (**d**) viable cells of *Chlorella vulgaris.* Changes in ROS in cells exposed to increasing concentrations of Ag NPs. (**e**) 50 nm Ag NPs; (**f**) 100 nm Ag NPs (Asterisks indicate significant differences from control values (*p* < 0.05); n = 3).

Comparison between the different sizes of Ag NPs and AgNO_3_ at 100 mg L^−1^ and 200 mg L^−1^ revealed that there was a significant difference (*p* < 0.05) between Ag NPs of 100 nm and AgNO_3_ compared to the Ag NPs of 50 nm at 100 mg L^−1^ (Figure 5a). The same results were obtained at 200 mg L^−1^ (Figure 5b), suggesting that the inhibitory effect is both size and concentration dependent.

### 3.4. Determination of Chlorophyll a and Viable Cells Using Silver Nitrate (AgNO_3_)

Chlorophyll *a* and viable cell concentrations for *Chlorella vulgaris* were evaluated using AgNO_3_ as a source of Ag^+^ ions. Ag^+^ ions showed a considerable reduction in both chlorophyll *a* concentration and viable cells at 100 and 200 mg L^−1^ compared to the control (Figure 5c,d). Control cultures displayed an increase in viable cell concentration from 11.1 × 10^5^ cell mL^−1^ at 24 h to 77.2 × 10^5^ cell mL^−1^ at 96 h. In contrast, there was a decline from 9.44 × 10^5^ cell mL^−1^ at 24 h to 6.16 × 10^5^ cell mL^−1^ at 96 h using 100 mg L^−1^ AgNO_3_, and from 9.69 × 10^5^ cell mL^−1^ at 24 h to 6.46 × 10^5^ cell mL^−1^ at 96 h using 200 mg L^−1^ AgNO_3_. A great decline was also observed in chlorophyll *a* concentration during the investigation period (Figure 5c). No precipitates were detected in the solution under the used conditions.

In aquatic environments, it is more plausible that oxidation of Ag NPs’ surface will occur by dissolved oxygen in water, and silver ions will be liberated [44]. Navarro et al. [4,32] and Leclerc and Wilkinson [45] found that toxicity is linked to Ag^+^ ions released from NPs. NPs that are soluble in the medium and release free ions are believed to be as the most familiar mechanisms of toxicity for numerous NPs [46] and Ag^+^ is identified to be one of the most phytotoxic metal ions [47] due to its cationic character and its powerful association with a variety of ligands in natural waters. The amount of ligands and their strength will define the toxicity of Ag^+^ ions [47]. Johari et al. [48] found that the toxicity of AgNO_3_ was significantly higher than Ag NPs at different saltwater medium. This could explain the current study results. However, more investigations are crucial to assess and compare between Ag^+^ ions and Ag NPs effects.

### 3.5. Reactive Oxygen Species (ROS) Production

The increased production of ROS was observed only after 48 h of incubation for both Ag NPs sizes. The belated increase can be linked to the presence of a cell wall on the *Chlorella vulgaris* [28]. Although ROS formation was observed to increase at the end of 96 h incubation, the algae treated with 50 nm Ag NPs showed a sharper increase in ROS formation at 200 mg L^−1^. The larger Ag NPs (100 nm) led to a higher rate of ROS production. On the whole, it was found that the concentration of Ag NPs has an effect on ROS formation, even though the overall fluorescence intensity was about two-fold for 100 nm Ag NPs-treated algae (Figure 5e,f).

The rates were observed to change in the same trend as the changes in cell viability and chlorophyll *a* content analyses. For instance, for 50 nm Ag NPs-treated cells, ROS production at the 96 h time point was more significant for only 100 and 200 mg L^−1^, whereas 100 nm Ag NP-treated cells at 50, 100 and 200 mg L^−1^ showed a higher increase. Similarly, chlorophyll *a* content significantly decreased when the cells were treated with 100 nm Ag NPs at 50, 100, and 200 mg L^−1^ concentrations. The correlation between both change rates can be originating from the ROS production capability of chloroplasts [49].

### 3.6. TEM Analyses

**Figure 6:**
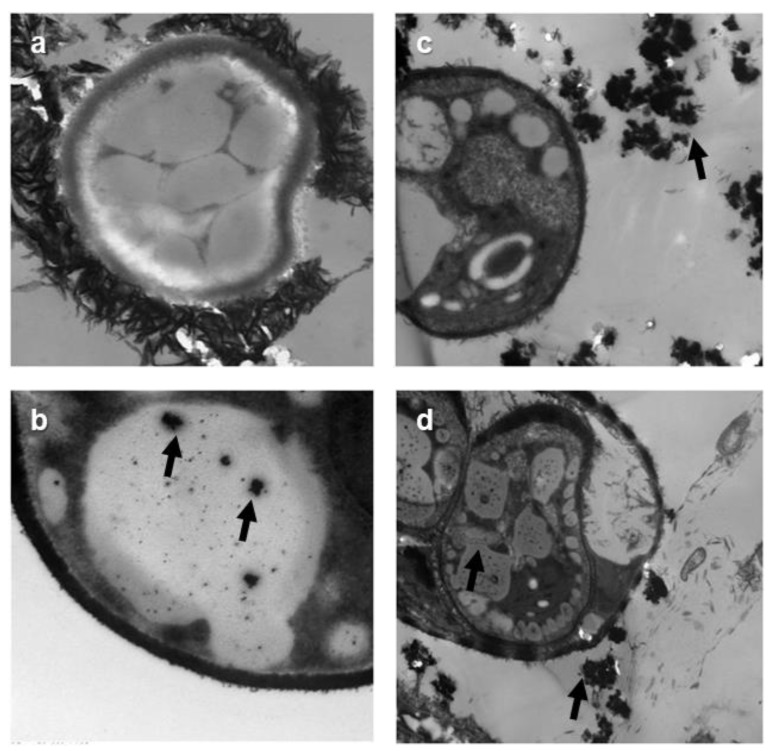
Transmission Electron Microscopy (TEM images for cells exposed to different concentration of Ag NPs. (**a**) control; (**b**) 50 nm, 200 mg L^−1^; (**c**,**d**) 100 nm, 200 mg L^−1^ (Arrows indicate the aggregates of Ag NPs in the culture medium and inside cells).

TEM analyses revealed that Ag NPs were present inside algal cells. Electron-dense precipitates can be observed in the vacuoles only at 200 mg L^−1^. Some Ag NP aggregates can also be seen in the medium (Figure 6). The aggregates were larger for larger Ag NPs. Sendra et al. [43] found that the attachment of Ag NPs on the surfaces of cells and the presence of Ag NPs inside cells seem to define toxicity to the aquatic organisms. Concerning the presence of large aggregates of Ag NPs observed in the culture media, it is very significant to note that large aggregates will increase with an increase in NaCl concentration [35].

In the present study, Ag NPs were purchased from (American Elements, Weyburn Ave., Los Angeles USA), and, according to the manufacturer, the diameters of NPs are 50 and 100 nm. However, the calculated crystallite size values were 21 and 32 nm for 50 and 100 nm Ag NPs, respectively. The small crystallites (21 and 32 nm) may explain the occurrence of Ag NPs inside the algal cells and stored inside vacuoles, as shown in Figure 6.

The fact that NPs were found inside algal cells could be due to either the Ag NPs entering the cells in the form of Ag^+^ ions (released from Ag NPs), or in a neutral AgCl form (formed from complexation with the Cl^−^ ions contained in the seawater medium). Sendra et al. [43] found that the main species that can be formed in marine water are AgCl_2_^−^ and AgCl_3_^2−^. The chemical species present in marine water are not bio-accessible to microalgae in comparison to freshwater culture media [43]. Under the present experimental conditions, the formation of neutral silver chloride complexes also come into play as shown by Reinfelder and Change [50], who reported that neutral silver chloride complexes could enter marine microalgae cells through passive diffusion through membranes. Leonardo et al. [51] have shown that Ag NPs were formed in the cells after the reduction of Ag^+^ inside the cells. They observed that Ag NPs formation inside the cells of a green microalga after exposure to Ag^+^ ions. They showed that Ag, at low concentrations inside the cells, first remains in the cell cytosol in an Ag (+I) form. When Ag concentration increases, Ag (+I) is reduced and forms Ag(0) NPs on different cell structures and in different organelles, particularly along the plasma membrane, in the chloroplasts, and in mitochondria. NPs can similarly enter into the cells via ion channels and transport proteins. Furthermore, endocytosis is an extra means for the internalization of NPs [52]. It should be noted that some microalgae could also have the ability to yield internal NPs from dissolved metals [35]. This scenario should be further explored. It has been identified now that microalgae are able to accumulate metals in internal granules as an effective way for detoxification [53].

It has been identified that Ag NPs suspension in ion-rich liquid solution, for example, brackish and saltwater environments, frequently agglomerates, which leads to changes in their surface area, charge, and size in comparison to the as-synthesized particles [54]. NPs are more likely to aggregate in seawater than in freshwater [55]. Oukarrum et al. [28] reported the development of large aggregates of Ag NPs in seawater growth medium used for the cultivation of marine microalgae *D. tertiolecta*. Thus, NP aggregation in sweater medium could have contributed to the effects of Ag NPs on *Chlorella vulgaris*.

## 4. Conclusions

On the whole, whether the toxicity of Ag NPs is due to the nanosize structure or to the released silver ions has been a contentious topic for a lengthy time, and the conclusion seems to be contingent on the features of the Ag NPs considered and/or the investigation setup (Burchardt et al. 2012). Our results demonstrated that Ag NPs are more toxic to the marine microalga, *Chlorella vulgaris*, for the larger size of Ag NPs (100 nm) and at the higher concentrations (100 and 200 mg L^−1^) of both sizes of Ag NPs. Solubility, nanoparticle size, and the degree of aggregation are significant controls on the toxicity of nanoparticles. The inhibitory effect was size and concentration-dependent, and the degree of aggregation of NPs increased with both size and concentration. Ag NPs were seen inside cells and accumulated inside vacuoles, and large Ag NPs aggregates were detected in the culture seawater medium. A decrease in viable cells, reduction in chlorophyll *a* concentration, and an increase in ROS formation are a comprehensive and established endpoint for Ag NPs toxicity. Further investigations dealing with different Ag NPs sizes, model organisms, and different experimental conditions should be considered. The effect of the culture medium (i.e., freshwater or seawater) should be further investigated.
